# AACVD of Cu_3_N on Al_2_O_3_ Using CuCl_2_ and NH_3_

**DOI:** 10.3390/ma15248966

**Published:** 2022-12-15

**Authors:** Matthew Zervos

**Affiliations:** Nanostructured Materials and Devices Laboratory, School of Engineering, University of Cyprus, P.O. Box 20537, Nicosia 1678, Cyprus; zervos@ucy.ac.cy

**Keywords:** copper nitride, chemical vapor deposition, structural, electrical properties

## Abstract

Cu_3_N has been grown on m-Al_2_O_3_ by aerosol-assisted chemical vapor deposition using 0.1 M CuCl_2_ in CH_3_CH_2_OH under an excess of NH_3_ at 600 °C, which led to the deposition of Cu that was subsequently converted into Cu_3_N under NH_3_: O_2_ at 400 °C in a two-step process without exposure to the ambient. The reaction of CuCl_2_ with an excess of NH_3_ did not lead to the growth of Cu_3_N, which is different to the case of halide vapor phase epitaxy of III-V semiconductors. The Cu_3_N layers obtained in this way had an anti-ReO_3_ cubic crystal structure with a lattice constant of 3.8 Å and were found to be persistently n-type, with a room temperature carrier density of n = 2 × 10^16^ cm^−3^ and mobility of µ_n_ = 32 cm^2^/Vs. The surface depletion, calculated in the effective mass approximation, was found to extend over ~0.15 µm by considering a surface barrier height of ϕ_B_ = 0.4 eV related to the formation of native Cu_2_O.

## 1. Introduction

Cu_3_N is a novel semiconductor in which crystal imperfections such as Cu interstitials (Cu_i_) and nitrogen vacancies (V_N_) give rise to states that are energetically located inside or very close to the conduction and valence bands, respectively [1], but do not give rise to any mid-gap states. Consequently, it has been suggested to be suitable as a solar cell absorber in view of the fact that it has an indirect energy band gap of ~1.0 eV [2], but also due to the fact that n- and p-type doping are possible. However, despite the fact that Cu_3_N has been described as a defect-tolerant semiconductor, so far no one has fabricated a working p-n junction solar cell using Cu_3_N. In the past, Chen et al. [3] fabricated a Cu_3_N p-n homojunction on indium tin oxide, and Yee et al. [1] fabricated an Al: ZnO/ZnS/Cu_3_N p-n heterojunction, both of which exhibited rectifying behavior but no photogenerated current. This has been attributed to the large concentration of Cu_i_ defects, which capture electrons and result into substantial Shockley–Read–Hall recombination and quenching of the steady-state minority carrier concentration under illumination. In other words, crystal imperfections such as V_N_ and Cu_i_ can still reduce the minority carrier lifetime and prevent the extraction of photogenerated electron–hole pairs in Cu_3_N. Nevertheless, Cu_3_N has been used successfully for energy storage as it has a cubic anti-ReO_3_ crystal structure, belonging to the Pm3m space group (number 221) with a lattice constant of 3.8 Å [4], and a vacant body center that can readily act as a host for Li ions in batteries [5]. Cu_3_N has been obtained by many different methods including reactive sputtering [6], molecular beam epitaxy [7], atomic layer deposition [8,9] and pulsed laser deposition [10,11]. Recently, we converted Cu into Cu_3_N under NH_3_: O_2_ between 400 °C and 600 °C, and observed distinct spectral features and maxima in differential transmission at 500 nm (≡2.48 eV), 550 nm (≡2.25 eV), 630 nm (≡1.97 eV) and 670 nm (≡1.85 eV) on a ps time scale by ultrafast pump–probe spectroscopy (UPPS) [12]. These correspond to the M and R direct energy band gaps of bulk-relaxed and strained Cu_3_N in excellent agreement with density functional theory (DFT) calculations of the electronic band structure [12]. This observation of the M and R direct energy band gaps in fact confirmed that Cu_3_N has a clean energy band gap. More recently, we also showed that iodine-doped Cu_3_N, i.e., I: Cu_3_N, is a p-type semiconductor and that the extensive incorporation of I in Cu_3_N can be used to convert Cu_3_N into γ-CuI, which is a p-type transparent semiconductor that was used in conjunction with n-type Cu_3_N, for the fabrication of a γ-CuI/TiO_2_/Cu_3_N p-n heterojunction that exhibited rectifying current–voltage characteristics [13]. In the past, most have focused on n-type doping of Cu_3_N, such as Gao et al. [14], who showed that the incorporation of Zn resulted into n-type Cu_3_N and increased the carrier density from n = 10^17^ to 10^21^ cm^−3^ with a resistivity of 10^−3^ Ω cm. In contrast, only a few have considered p-type doping of Cu_3_N, such as Matsuzaki et al. [15], who used NF_3_ for the growth of F-doped p-type Cu_3_N. The ability to obtain p-type Cu_3_N is important, of course, for the realization of p-n junctions, but it should be noted that I-VII γ-cuprous halides such as γ-CuCl, CuBr and CuI are p-type transparent semiconductors with a zinc blende crystal structure and direct energy band-gaps of 3.3, 2.9 and 2.95 eV, respectively. In addition, they have lattice constants close to that of Si [16] and may be readily combined with Cu_3_N for the realization of novel cuprous electronic and optoelectronic devices.

Here Cu_3_N has been obtained by aerosol-assisted chemical vapor deposition (AACVD) using CuCl_2_ in CH_3_CH_2_OH and NH_3_. AACVD is a low-cost growth method [17] that has been used for the growth of a broad range of semiconductors [18] including III-V semiconductors such as InN, GaN and In_x_Ga_1−x_N [19]. The growth of these III-Vs is carried out using anhydrous N_2_ and NH_3_, i.e., O_2_ and H_2_O are generally avoided and eliminated. In the past, McInnes et al. [19] used 0.1M GaCl_3_ and 0.1M InCl_3_ in acetonitrile (CH_3_CN), N_2_ as carrier gas and anhydrous NH_3_ to grow In_x_Ga_1−x_N. The total flow rate through the 0.1 M GaCl_3_ and 0.1 M InCl_3_ solutions was maintained at 529 mL min^−1^, and anhydrous NH_3_ was used at a high flow rate of 862 mL min^−1^, which assisted in promoting the formation of smaller droplets whilst also ensuring an excess of NH_3_ for the deposition of In_x_Ga_1-x_N. The deposition was carried out at 600 °C, which exceeds the melting point of both GaCl_3_ and InCl_3_ and gave layers with a thickness of ~2 μm. Both GaCl_3_ and InCl_3_ react directly with NH_3_, leading to the deposition of GaN and InN, respectively, while CH_3_CN [20] breaks into HCN and CH_4_ at elevated temperatures [21,22].

AACVD has also been used for the growth of Cu_3_N by Yamaguchi et al. [23], who obtained Cu_3_N on α-Al_2_O_3_ by AACVD at 300 °C using copper (II) acetylacetonate Cu(O_2_C_5_H_7_)_2_ that was dissolved in aqueous NH_3_. No CuO or Cu_2_O was detected in the Cu_3_N despite the fact that Cu(O_2_C_5_H_7_)_2_ was used in aqueous NH_3_. Others such as Park et al. [8] used metal organic sources of copper such as C_14_H_32_CuN_2_O_2_, which contains oxygen in conjunction with NH_3_ for the atomic layer deposition of Cu_3_N, but metal organic sources are expensive [9].

No one has previously attempted to grow Cu_3_N using CuCl_2_ and NH_3_ by AACVD or tried to grow Cu_3_N on m-Al_2_O_3_, which is ideally suited for the growth of cubic and tetragonal crystals. It is found that the reaction of CuCl_2_ with NH_3_ will not give Cu_3_N as in the case of halide vapor phase epitaxy (HVPE) of III-V semiconductors such as In_x_Ga_1−x_N. In contrast, the reaction of CuCl_2_ with an excess of NH_3_ resulted into the deposition of polycrystalline Cu layers consisting of oriented grains on m-Al_2_O_3_, which have a higher crystal quality compared to Cu obtained by sputtering [12] or electron beam evaporation [15] used previously to obtain Cu_3_N under NH_3_: O_2_. Consequently, the Cu layers on m-Al_2_O_3_ obtained via the reduction of CuCl_2_ under NH_3_ at elevated temperatures were converted into cubic Cu_3_N under NH_3_: O_2_ at a lower temperature without exposure of the Cu to the ambient. The Cu_3_N layers on m-Al_2_O_3_ have an anti-ReO_3_ cubic crystal structure with a lattice constant of 3.8 Å and are n-type with carrier density n = 2 × 10^16^ cm^−3^ and mobility µ_n_ = 32 cm^2^/Vs at room temperature. The electrical properties are described in conjunction with theoretical calculations of the conduction band potential profile, surface band bending and depletion in the effective mass approximation.

## 2. Materials and Methods

**Figure 1:**
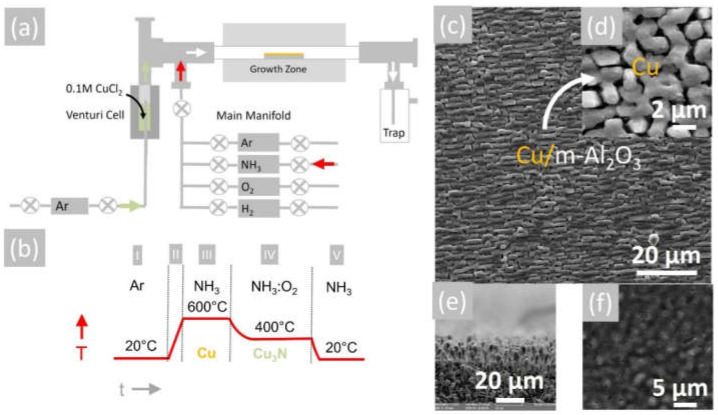
(**a**) Schematic of 1″ AACVD hot wall-reactor; (**b**) temperature–time profile for the deposition of Cu and conversion to Cu_3_N (**c**); (**d**) SEM image of Cu on m-Al_2_O_3_ obtained at 600 °C showing ordering of the grains; (**e**) side view of SEM image of Cu on m-Al_2_O_3_ obtained at 600 °C showing columnar growth; (**f**) SEM image of Cu_3_N obtained from Cu under NH_3_: O_2_ at 400 °C.

Initially, 1.34 mg of CuCl_2_ (Aldrich 99.999%, 134.45 gmol^−1^), which has a rusty-brown color, was dissolved in 100 mL ethanol CH_3_CH_2_OH and stirred at 1000 rpm for 10 min at room temperature. CuCl_2_ is soluble in water (75 g/100 mL H_2_O at 25 °C) and ethanol (53 g/100 mL CH_3_CH_2_OH at 25 °C) but less so in acetonitrile CH_3_CN (1.6 g/100 mL at 20 °C). In contrast, CuCl has a considerably lesser solubility than that of CuCl_2_. More specifically, CuCl is slightly soluble in water (0.0047 g/100 mL H_2_O at 20 °C) and insoluble in ethanol CH_3_CH_2_OH and acetone (CH_3_)_2_CO. In order to obtain a satisfactory growth rate, a 0.1 M solution of CuCl_2_ in CH_3_CH_2_OH was prepared that has a dark green color due to the (CuCl_4_)^2−^ ions that are yellow and Cu^+2^ ions that are blue. The 0.1 M CuCl_2_ liquid precursor was turned into a mist using a Venturi nebulizer and Ar as a carrier gas. Square samples of ~8 mm × 8 mm m-Al_2_O_3_ with a thickness of ~0.3 mm were cleaned sequentially in trichloroethylene, methanol, acetone and isopropanol at 80 °C, after which they were rinsed in deionized water at 20 °C and dried with nitrogen, followed by a dehydration bake at 120 °C. The clean m-Al_2_O_3_ was loaded in a quartz boat that was positioned at the center of a 1” hot wall, single zone AACVD reactor, capable of reaching 1100 °C that was fed by a manifold consisting of four mass flow controllers connected to Ar, NH_3_, O_2_ and H_2_ and a separate side manifold for controlling the flow of Ar through the Venturi nebulizer, as shown in Figure 1a. The reactor was purged with 1000 mL/min of Ar for 10 min from the main manifold, after which the temperature was ramped at 30 °C/min under a flow of 90 mL/min Ar: 10 mL/min H_2_ at one atmosphere. Upon reaching 600 °C, the flow of Ar: H_2_ was interrupted and a flow of 800 mL/min NH_3_ was initiated, while at the same time a flow of 1000 mL/min Ar was established through the nebulizer. A visible flow of the aerosol was observed on the upstream side that was maintained for 30 min. Subsequently, the flow of Ar through the Venturi nebulizer was interrupted and the reactor allowed to cool down to 400 °C under a flow of 300 mL min^−1^ NH_3_. Upon reaching 400 °C, the Cu was converted into Cu_3_N under a flow of 300 mL min^−1^ NH_3_ and 15 mL min^−1^ O_2_ for 30 min. At the end of the growth period, cool down took place under a flow of 300 mL/min NH_3_ supplied from the main manifold until the temperature fell below 100 °C. A typical temperature–time profile is shown in Figure 1b. The Cu_3_N layers were removed after purging with 1000 mL min^−1^ of Ar at room temperature and were stored in a desiccator under vacuum.

The morphology and crystal structure of the Cu_3_N layers were determined by scanning electron microscopy (SEM) and X-ray diffraction (XRD). The carrier density and mobility of the Cu_3_N layers were determined by the Hall effect in the van der Pauw configuration by using a Keithley 2635A constant current source in conjunction with a Keithley 2182 voltmeter controlled by LabView. 

## 3. Results and Discussion

**Figure 2:**
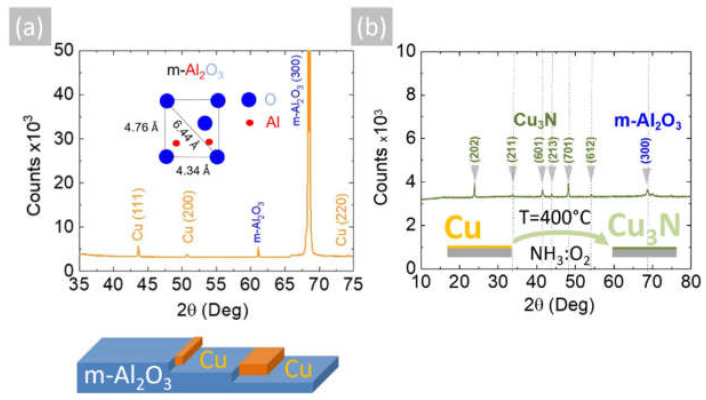
(**a**) XRD of Cu on m-Al_2_O_3_ obtained at 600 °C showing peaks belonging to Cu and m-Al_2_O_3_; inset shows the oxygen-terminated surface of m-Al_2_O_3_; (**b**) XRD of Cu_3_N obtained from Cu under NH_3_: O_2_ at 400 °C showing the peaks belonging to the anti-ReO_3_ cubic crystal structure of Cu_3_N and m-Al_2_O_3_.

The reaction of CuCl_2_ in CH_3_CH_2_OH with an excess of NH_3_ did not lead to the direct deposition of Cu_3_N, as in the case of HVPE of III-V semiconductors such as In_x_Ga_1−x_N, but resulted into the deposition of metallic Cu on m-Al_2_O_3_ that had a shiny, reflective surface and metallic conductivity. A typical SEM image of the Cu layer obtained on m-Al_2_O_3_ at 600 °C is shown in Figure 1c, from which one may observe that the Cu layer is polycrystalline and consists of grains oriented along a single direction. A higher magnification image is also shown in Figure 1d, from which it is evident that the grains have sizes of ~5 µm, while a side view of the Cu on m-Al_2_O_3_ is shown in Figure 1e, showing that columnar growth occurs. The epitaxial growth of Cu on c-Al_2_O_3_ and a-Al_2_O_3_ has been investigated extensively for the growth of high-quality graphene [24,25], but only a few have considered the growth of Cu on m-Al_2_O_3_ [26]. The deposition of Cu on m-Al_2_O_3_, which contains grooves or steps along specific crystallographic directions, as shown in Figure 2a, will lead to instabilities and ruptures of the Cu layer at elevated temperatures [27]. These ruptures occur at high curvature sites, i.e., peaks and ridges, which act as retracting edges leading to a net flux of atoms away from the high positive curvature regions. For sufficiently thin layers, this process will lead to a self-assembly of the Cu grains along a specific direction [28].

**Figure 3:**
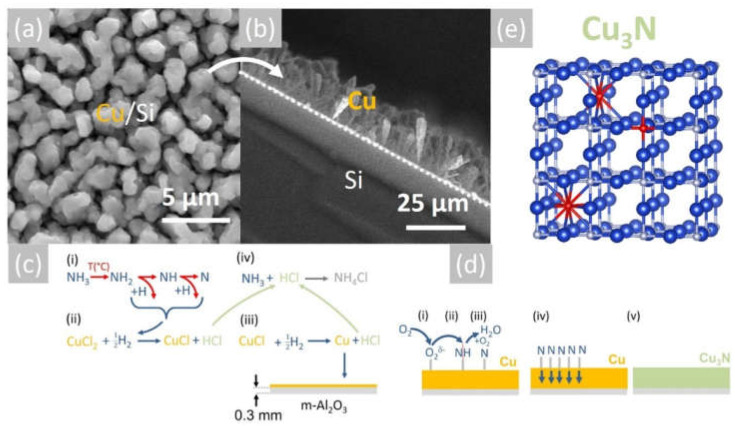
(**a**) SEM image of Cu on Si obtained at 600 °C and (**b**) section showing the formation of Cu rods on Si; (**c**) reaction mechanism for the deposition of Cu and (**d**) reaction mechanism for the conversion of Cu into Cu_3_N obtained under NH_3_: O_2_; (**e**) stick and ball model of the anti-ReO_3_ cubic crystal structure of Cu_3_N; large blue spheres (Cu), small grey spheres (N) and red spheres (O).

The Cu layers exhibited clear peaks in the XRD, as shown in Figure 2a, corresponding to the face-centered cubic (fcc) crystal structure of Cu with a lattice constant of 3.6 Å. No peaks belonging to CuO, Cu_4_O_3_ or Cu_2_O are observed in Figure 2a. Likewise shown are the peaks corresponding to the underlying m-Al_2_O_3_, which has an oxygen-terminated surface with tetragonal crystal symmetry that is suitable for the epitaxial growth of semiconductors with a cubic crystal structure. It is worthwhile to point out that the deposition of Cu on n-type Si (001) resulted in columnar growth, as shown in Figure 3a,b. The Cu pillars have a height of ~20 µm, but they are not ordered in any way. No Cu_3_N was obtained under an excess of NH_3_ by varying the temperature between 300 °C and 800 °C. Instead, the reaction of CuCl_2_ in CH_3_CH_2_OH with NH_3_ always led to the deposition of Cu on m-Al_2_O_3_, which occurs via the reduction of CuCl_2_ to CuCl and then into Cu by the H_2_ evolving from NH_3_.

More specifically, the 0.1 M solution of CuCl_2_ is initially converted into a mist of liquid drops and mixed with NH_3_, which is soluble in CH_3_CH_2_OH [29]. Subsequently, the liquid drops are vaporized at an elevated temperature, and the CH_3_CH_2_OH gives C_2_H_4_ and H_2_O according to the reaction C_2_H_5_OH → C_2_H_4_ + H_2_O. No carbon is released from the pyrolysis of C_2_H_4_ between 500 °C and 800 °C [30]. Upon vaporization, CuCl_2_, which has a melting point of 498 °C, will be reduced to CuCl, which has an even lower melting point of 423 °C [31], and finally into metallic Cu by the H_2_ evolving from the breakdown of NH_3_. Before elaborating further, it is useful to note that the thermal breakdown of NH_3_ into N_2_ and H_2_ was investigated as early as 1905 by White et al. [32], who showed that it depends on the gas flow, i.e., residence time as well as the temperature. In particular, White et al. [32] showed that a flow of 200 mL/min NH_3_ resulted in a dissociation of 5% NH_3_ at 600 °C and 10% at 700 °C. However, the breakdown of NH_3_ is also promoted catalytically by the deposited Cu at elevated temperatures [33]. In other words, the Cu deposited on the m-Al_2_O_3_ will participate actively in the dissociation of NH_3_ near the surface, thereby further promoting the reduction of CuCl_2_ and deposition of Cu, which has a melting point of 1085 °C. A schematic representation of the proposed reaction mechanism is shown in Figure 3c. For completeness, it must also be pointed out that the NH_3_ will react with CH_3_CH_2_OH and give ethylamine (CH_3_CH_2_NH_2_) and acetonitrile (CH_3_CN), which have boiling points of 20 °C and 82 °C, respectively. CH_3_CH_2_NH_2_ and CH_3_CN will dissociate into HCN and CH_4_ depending on the temperature and residence time, but they are not expected to influence the overall reaction governing the deposition of Cu. It is also important to mention that the Cu will tend to react with H_2_O supplied from the CH_3_CH_2_OH and give CuO and Cu_2_O. However, no oxides are detected in Figure 2a, so it is very likely that they are reduced to metallic Cu due to the H_2_ evolving from the NH_3_ over the Cu. This is consistent with the findings of Kim et al. [34], who showed that CuO is converted into metallic Cu under an excess of H_2_ without the formation of intermediate Cu_4_O_3_ or Cu_2_O.

In short, CuCl_2_ is reduced to CuCl and then into Cu by the H_2_ evolving from NH_3_, according to: CuCl_2_ + H_2_ → CuCl + 2HCl and 2CuCl + H_2_ → 2Cu + 2HCl. The HCl reacted in turn with the excess NH_3_, giving NH_4_Cl, i.e., NH_3_ + HCl → NH_4_Cl, which solidified below its sublimation temperature, i.e., ~340 °C near the cool end of the reactor, very similar to what occurs during conventional HVPE of III-Vs.

**Figure 4:**
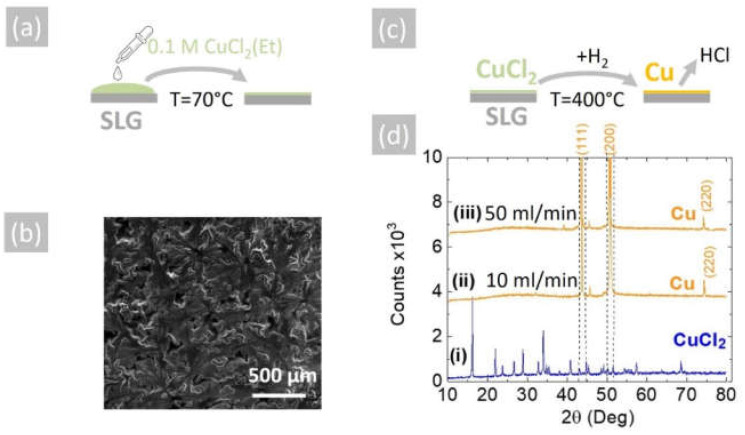
(**a**) Schematic illustration of drop-casting a 0.1 M CuCl_2_ (Et) solution on SLG at 70 °C to aid the evaporation of the ethanol; (**b**) SEM image of the CuCl_2_ on SLG, which had a light green color; (**c**) schematic of conversion of CuCl_2_ into Cu under H_2_ at 400 °C; (**d**) XRD of (i) CuCl_2_ as-deposited on SLG, (ii) Cu obtained from CuCl_2_ under 10 mL/min H_2_ and (iii) 50 mL/min H_2_. All of the CuCl_2_ peaks vanish, and the peaks belonging to Cu do not overlap with those of CuCl_2_, as shown with the aid of the broken vertical lines.

The reduction of CuCl_2_ into Cu may also be achieved by using H_2_ as opposed to NH_3_. In order to show this, the 0.1 M solution of CuCl_2_ in CH_3_CH_2_OH was used to deposit a layer of CuCl_2_ on 15 mm × 30 mm soda lime glass (SLG) slides by drop-casting, as shown in Figure 4a. The CuCl_2_ layer had a light green color and good uniformity, and a typical SEM image is shown in Figure 4b. This was converted into Cu under a flow of (i) 10 and (ii) 50 mL.min^−1^ pure H_2_ at 400 °C for 30 min, as shown schematically in Figure 4c. The CuCl_2_ as-deposited on SLG displayed a crystalline structure and multiple peaks in the XRD, as shown in Figure 4d, but all the peaks were eliminated after the reduction of the CuCl_2_ into Cu.

The Cu deposited on m-Al_2_O_3_ at 600 °C by AACVD using CuCl_2_ and NH_3_ has a higher crystal quality compared to the Cu obtained by sputtering, which was nonetheless successfully converted into crystalline Cu_3_N under a flow of 300 mL/min NH_3_ and 15 mL/min O_2_ between 400 °C and 600 °C, as shown previously [12]. The Cu_3_N obtained in this way had an anti-ReO_3_ cubic crystal structure, and we observed the M and R direct energy band gaps of Cu_3_N by UPPS in excellent agreement with DFT calculations of the electronic structure, confirming that it has a clean energy gap [12]. Consequently, the polycrystalline Cu layer that was obtained by AACVD on m-Al_2_O_3_ at 600 °C was converted into Cu_3_N under a flow of 300 mL/min NH_3_ and 15 mL/min O_2_ at 400 °C. The Cu_3_N had an olive-green-like color, and a typical SEM image of the Cu_3_N layer on m-Al_2_O_3_ is shown in Figure 1f. This exhibited peaks in the XRD, as shown in Figure 2b, corresponding to the anti-ReO_3_ cubic crystal structure of Cu_3_N with a lattice constant of 3.8 Å.

The reaction of Cu with NH_3_ containing O_2_ and the formation of Cu_3_N can be understood by considering the catalytic oxidation of NH_3_ by O_2_ in the presence of a catalyst, e.g., Cu, Pt, etc., at elevated temperatures, as described by Carley et al. [35], who investigated the catalytic reactivity of Cu (110) metal surfaces with coadsorbed NH_3_ and O_2_. More specifically, Carley et al. [35] proposed that the oxidation of NH_3_ leads to the formation of a stabilized N monolayer on the Cu metal surface, which in turn is responsible for the conversion of the bulk Cu layer into Cu_3_N. It should be noted that the reaction of NH_3_ with O_2_ also gives H_2_O according to the reaction NH_3_ + O_2_ → NO + H_2_O, which was observed to condense near the cool end of the reactor upon increasing the gas flow of O_2_. The reaction mechanism of the conversion of Cu into Cu_3_N is depicted schematically in Figure 3d. No Cu_3_N was obtained from Cu by using only NH_3_, in accordance with Matsuzaki et al. [15]. Moreover, no CuO or Cu_2_O peaks are detected in Figure 2b, but Cu_2_O will nevertheless form as native oxide on the surface of the Cu_3_N upon exposure to the ambient, as we have shown previously by using Raman spectroscopy [36]. Before considering the electrical properties of the Cu_3_N layers, it is useful to point out that the reaction of CuCl_2_ with a smaller flow of 100 mL/min NH_3_ at 600 °C mainly led to the deposition of Cu_2_O, not Cu_3_N.

In order to measure the Hall effect, Ag ohmic contacts were deposited at the four corners of the Cu_3_N layers on m-Al_2_O_3_. It has been shown that Au, Ag and Cu in Cu_3_N give rise to a semiconductor-to-metal transition and remarkably constant electrical resistivity over a very broad range of temperatures [37]. Consequently Ag, Au and Cu may be used for the formation of ohmic contacts on Cu_3_N, and in the past, we have shown that Au and Ag deposited on n-type Cu_3_N results in the formation of contacts with linear IVs [13]. The Cu_3_N layers on m-Al_2_O_3_ were found to be n-type and had room temperature carrier densities of 2 × 10^16^ cm^−3^ with a maximum mobility of 32 cm^2^/Vs. The Cu_3_N layers are n-type as they are Cu-rich, but also due to the fact that oxygen may be included in the Cu_3_N by the preferential formation of interstitial oxygen (O_i_) that acts as donors, not as acceptors [36]. Furthermore, the Cu_3_N layers obtained here were found to be persistently n-type, and the carrier density and mobility did not exhibit any changes upon illumination with light of λ = 450 nm under ambient conditions. In other words, the n-type Cu_3_N layers did not exhibit any photoconductivity, which may be attributed to recombination via Cu_i_ and V_N_ states, in accordance with Yee et al. [1].

It is worthwhile pointing out here that Matsuzaki et al. [15] showed that epitaxial Cu_3_N layers with a thickness of 25 nm on SrTiO_3_ were p-type, due to the upward surface band bending mediated by the chemisorption of O_2_^−^, but switched to n-type upon exposure to ultraviolet (UV) light and reverted back to p-type after terminating the irradiation. In contrast, they observed that the Cu_3_N layers remained n-type after exposure to UV light under vacuum, confirming that the adsorbed O_2_^−^ is responsible for the surface inversion observed under ambient conditions in air. However, the epitaxial Cu_3_N layers of Matsuzaki et al. [15] were found to be persistently n-type under ambient conditions, with a carrier density of the order of 10^14^ cm^−3^ and mobility of 100 cm^2^/Vs after annealing under NH_3_ between 125 and 350 °C, suggesting a change in the composition of the surface and overall band bending. The Cu_3_N layers obtained here were found to be persistently n-type and had a room temperature carrier density of 2 × 10^16^ cm^−3^, perhaps due to the fact that after the conversion of Cu into Cu_3_N under NH_3_: O_2_, the flow of NH_3_ was maintained for at least 30 min until the temperature fell well below 100 °C.

**Figure 5:**
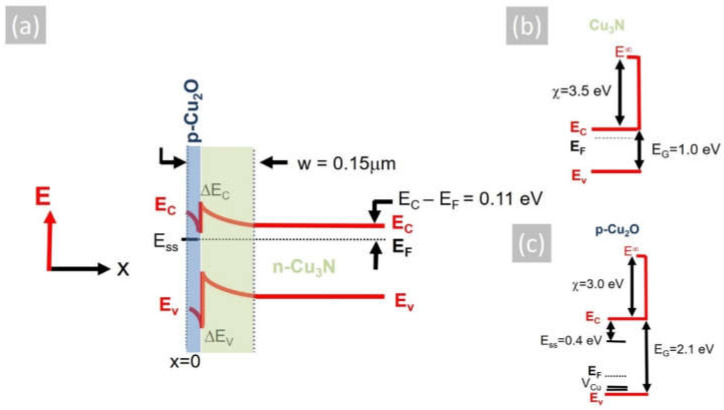
(**a**) Energy band potential profile of a Cu_2_O/Cu_3_N p-n heterojunction consisting of n-type Cu_3_N and the native, surface p-type Cu_2_O, where the Fermi level (E_F_) is pinned at the surface states (E_ss_) residing ~0.4 eV below the conduction band edge of Cu_2_O; also shown are the conduction (ΔE_C_) and valence (ΔE_V_) band discontinuities as well as the surface depletion w that extends 0.15 μm through the p-type Cu_2_O and into the n-type Cu_3_N. (**b**) Energy band diagram of n-type Cu_3_N showing the conduction (E_C_) and valence (E_V_) bands as well as the Fermi level (E_F_) and electron affinity (χ) of Cu_3_N, (**c**) same for Cu_2_O; also shown are the (V_Cu_) acceptor states, which reside above the top of the valence band, and the energetic position of the surface states (E_ss_).

However, the properties of Cu_3_N layers with a thickness of a few tens of nm will depend strongly on the properties of the surface but also the properties of the underlying substrate that is often overlooked. The Cu_3_N layers obtained here are persistently n-type with a carrier density of 2 × 10^16^ cm^−3^, most likely due to the fact that the thickness of the Cu_3_N layers is greater than 1 µm, so it is bulk-like and will not be strongly influenced by properties of the surface or underlying m-Al_2_O_3_. In thermodynamic equilibrium, the Fermi level (*E_F_*) with respect to the conduction band minimum (*E_C_*) away from the surface and deep in the bulk is determined from:

$$n=N_{C}e^{-\frac{E_{C}-E_{F}}{kT}},$$

where *N_C_* is the conduction band effective density of states, *k* is Boltzmann’s constant and T the temperature taken to be equal *T* = 300 K. The electron density is equal to *n* = 2 × 10^16^ cm^−3^, and the conduction band effective density of states in Cu_3_N is given by:

$$N_{C}=2{\left(\frac{2\pi m_{n}kT}{h^{2}}\right)}^{\frac{3}{2}},$$

where *m_n_* is the electron effective mass in Cu_3_N taken to be m_n_= 0.16 m_o_ [6], m_o_ is the free-electron mass and h is Planck’s constant. This gives *N_C_* = 1.6 × 10^24^ m^−3^ or 1.6 × 10^18^ cm^−3^, so *E_C_* − *E_F_* = 0.11 eV in the bulk where a flat band condition exists. On the other hand, the energetic position of the Fermi level with respect to the conduction band edge, i.e., *E_C_* − *E_F_*, at the surface is dependent on the local density and energetic position of any surface states that will be occupied by electrons, which in turn may pin the Fermi level at the surface. According to Navío et al. [38], the Fermi level at the surface of ultrathin Cu_3_N layers is pinned at the middle of the gap, which will give rise to a barrier height of ϕ_b_ = 0.5 eV. The surface depletion region will extend into the Cu_3_N, and the depletion width is:

$$w=\sqrt{\frac{2\epsilon_{s}\varphi_{b}}{eN_{D}}},$$

where *ε_S_* = *ε_R_ε_o_*, *ε_R_* is the static dielectric constant of Cu_3_N, *ε_o_* the permittivity of free space, *e* the electron charge and *N_D_* the donor density taken to be equal to *n* = 2 × 10^16^ cm^−3^. Considering that the static dielectric constant of Cu_3_N is *ε_R_* ~10 [39], the depletion width is found to be *w* = 0.16 µm, taking into account that the Fermi level at the surface of Cu_3_N layers is pinned at the middle of the gap, according to Navío et al. [38]. However, despite the fact that we did not detect any CuO or Cu_2_O in the XRD, a thin layer of Cu_2_O will exist on the surface of Cu_3_N. According to Hodby et al. [40], the Fermi level at the surface of Cu_2_O is pinned at states residing energetically in the upper half of the band gap ~0.4 eV below the conduction band edge. The native Cu_2_O layer of Cu_3_N is expected to have a thickness of only a few nm and will be completely depleted, so the depletion width taking ϕ_b_ = 0.4 eV is found to be *w* = 0.15 µm. The conduction band potential profile of the Cu_3_N layer including the native Cu_2_O layer at its surface is shown in Figure 5a, where the work function and electron affinity of Cu_3_N i.e., ϕ(Cu_3_N) = 5.0 eV and χ(Cu_3_N) = 3.5 eV [41] have been considered as well as the work function and electron affinity of Cu_2_O, i.e., ϕ(Cu_2_O) = 4.8 eV and χ(Cu_2_O) = 3.2 eV [42]. The formation of p-type Cu_2_O over the n-type Cu_3_N will lead to the confinement of photogenerated electron–hole pairs at the Cu_2_O/Cu_3_N heterojunction, which will inadvertently result into recombination via states at the interface, thereby suppressing the photoconductivity. This mechanism is different to that put forward by Yee et al. [1], who fabricated an Al: ZnO/ZnS/Cu_3_N p-n heterojunction that exhibited rectifying behavior but no photogenerated current, which was attributed to the large concentration of Cu_i_ defects that capture electrons and result in substantial Shockley–Read–Hall recombination and quenching of the steady-state minority carrier concentration under illumination. While it is possible that both mechanisms are responsible for the suppression of the photocurrent and photoconductivity in Cu_3_N, it is imperative that the surface recombination should be suppressed via the deposition of suitable layers that prevent the formation of Cu_2_O that was originally suggested to act as a suitable passivation layer for Cu_3_N, similar to that of SiO_2_ for Si p-n junction solar cells [2].

## 4. Conclusions

Cu_3_N layers have been grown on m-Al_2_O_3_ by aerosol-assisted chemical vapor deposition using 0.1 M CuCl_2_ in CH_3_CH_2_OH under an excess of NH_3_ at 600 °C, which resulted in the deposition of epitaxial Cu layers consisting of oriented grains with a face-centered cubic crystal structure that were subsequently converted into Cu_3_N under NH_3_: O_2_ at 400 °C in a two-step process without exposure to the ambient. The reaction of CuCl_2_ with an excess of NH_3_ did not give Cu_3_N, which is different to halide vapor phase epitaxy of III-V semiconductors such as In_x_Ga_1−x_N. The Cu_3_N layers obtained in this way have an anti-ReO_3_ cubic crystal structure and persistent room temperature carrier density of n = 2 × 10^16^ cm^−3^ and mobility of µ_n_ = 32 cm^2^/Vs, but they did not exhibit any photoconductivity due to recombination via surface states in the Cu_2_O or via indirect recombination via Cu_i_ defects, which capture electrons and result into substantial Shockley–Read–Hall recombination
